# Are green spaces healthier in spring? Examining seasonal variations in the association between urban green spaces and emotional well-being in Wuhan, China

**DOI:** 10.3389/fpubh.2026.1833150

**Published:** 2026-05-22

**Authors:** Zitong Xu, Zhiqi Zhang, Bo Li, Yiyang Wu, Yanfei Jia

**Affiliations:** 1 School of Architecture and Urban Planning, Huazhong University of Science and Technology, Wuhan, China; 2 Hubei Engineering and Technology Research Center of Urbanization, Wuhan, China; 3 School of Architecture, Tsinghua University, Beijing, China; 4 School of Computer Science & Technology, Huazhong University of Science and Technology, Wuhan, China

**Keywords:** negative emotions, nonlinear effects, season, spatial heterogeneity, urban green space

## Abstract

**Background:**

Urban green spaces (UGS) are widely recognized as environmental elements closely associated with emotional health. However, most existing studies rely on static or annual average indicators to examine associations between green spaces and mental health, while paying insufficient attention to seasonal dynamics, multidimensional UGS characteristics (including quantity, quality, and landscape patterns), and nonlinear and spatially heterogeneous associations with negative emotions. Clarifying these seasonally varying associations can provide a more practical basis for urban green space planning.

**Methods:**

Using geotagged Weibo data from Wuhan in 2022, we identified negative emotional expressions with a fine-tuned BERT model and constructed a population-standardized indicator of negative emotions. Based on remote sensing imagery, street view images, and POI data, we developed a multidimensional evaluation framework of UGS characteristics. To account for the particular social context of 2022, lockdown intensity was incorporated as a control variable in all models. We applied geographically weighted random forest (GWRF) and SHAP-based interpretation to examine seasonal associations between UGS characteristics and negative emotions, focusing on variable importance, nonlinear threshold patterns, and spatial heterogeneity across four seasons.

**Results:**

After population standardization of the negative emotion indicator and adjustment for lockdown intensity, negative emotions still showed clear seasonal fluctuations and significant spatial clustering. NDVI, green space accessibility, and green space openness were identified as key correlates of negative emotions. Among them, green space accessibility showed association with lower negative emotions in spring than in other seasons. Several major factors exhibited consistent nonlinear turning points across seasons, although the magnitudes and directions of their associations varied seasonally, with more pronounced changes in spring and milder changes in winter. In addition, associations between UGS characteristics and negative emotions displayed substantial spatial heterogeneity, while the broad spatial pattern remained similar across seasons.

**Conclusion:**

Associations between UGS characteristics and negative emotions are seasonally dynamic rather than constant throughout the year. In particular, spring may represent a priority period during which green space accessibility and usability are more strongly associated with lower negative emotions. These findings highlight the importance of incorporating seasonal dynamics into UGS research and provide evidence for more adaptive, context-sensitive green space planning.

## 1 Introduction

Mental health and emotional well-being have become major public health concerns worldwide and are increasingly recognized as essential components of sustainable urban development. Negative emotions, as an important manifestation of mental health-related states, can affect individuals’ quality of life and are closely linked to broader social and environmental conditions (1, 57). Among various intervention strategies, UGS have received growing attention because of their potential associations with mental health and emotional well-being (2, 3). UGS can influence mental health through both direct and indirect pathways. Firstly, exposure to green spaces offers multiple direct benefits, including alleviating negative emotions, extending the duration of sustained attention, providing restorative experiences, and relieving stress (4–6). Secondly, UGS can also affect mental health through indirect mechanisms, such as facilitating physical activity and promoting social interactions (7, 8).

However, UGS systems encompass diverse types, and their associations with mental health may depend on multiple dimensions, including green space quantity, accessibility, and landscape characteristics, which may operate through different pathways (3, 9). Previous research has predominantly assessed the mental health implications of UGS from an overhead perspective, mainly through quantity-related indicators (10). Such metrics, typically derived from remote sensing data, mainly include the NDVI, green space area, and vegetation coverage (11). However, such indicators focus primarily on the overall coverage of green spaces, and are insufficient in reflecting residents’ actual perception and experience of green spaces. Consequently, they cannot fully explain the variations in individual subjective feelings (56). To overcome these limitations, recent studies have adopted an eye-level perspective to quantify visible greenness from the pedestrian viewpoint. These measures are commonly derived from street view imagery and characterize green visibility from a pedestrian viewpoint; among them, the green view index (GVI) is one of the most widely applied indicators (12, 13). GVI primarily focuses on green space visibility and provides limited systematic assessment of green space quality, such as accessibility and openness. A growing body of evidence suggests that green space quality is a key factor modulating the effects of UGS on mental health. For example, Nguyen et al. (14)‘s systematic review pointed out that green space quality has stable associations with multiple types of health outcomes. Wood et al. (15) found that higher green space quality was associated with better positive mental health, while Lee et al. (16) further demonstrated that green space accessibility could significantly mitigate the occurrence of psychological problems. Therefore, it is necessary to integrate multiple UGS indicators, covering different dimensions from an aerial perspective to eye level, and from quantity to quality. A more detailed and comprehensive evaluation framework for UGS should be constructed to further clarify the relationship between UGS and mental health (17, 18).

Traditionally, mental health has been measured primarily using individual questionnaire surveys, which are often constrained by limited sample sizes and restricted spatiotemporal coverage. In recent years, the widespread application of social media data has provided a novel perspective for investigating the relationship between multidimensional urban green space characteristics and mental health. Compared with traditional questionnaire survey data, social media posts can provide continuous observations of psychological states over broader geographic scales and longer time spans (19). Based on this, existing studies have utilized social media data to conduct various mental health analyses, including assessments of public sentiment (20), perceived tranquility (21), and spatial patterns of well-being across geographic spaces (22–24). These emotions and subjective feelings are widely recognized as important indicators of mental health.

However, most of these studies overlook the temporal dynamics of mental health status, particularly the pronounced seasonal variations (25, 26). Most existing evidence relies on annual averages or long-term consolidated data to analyze the association between UGS and emotional well-being, implicitly assuming that these associations remain stable throughout the year (3). This assumption, however, may oversimplify the complex interplay between environmental conditions and human behaviors. Seasonal shifts influence vegetation phenology, climatic comfort, and residents’ outdoor activity patterns, thereby altering the levels of multidimensional urban green space characteristics and their corresponding emotional effects (26, 27). For instance, in seasons with favorable climates and lush vegetation, residents engage more frequently with green spaces. Conversely, extreme temperatures or inclement weather reduce outdoor activities, thereby diminishing the benefits derived from green space accessibility (28). Therefore, it is necessary to further examine how the associations between emotional well-being and multidimensional UGS characteristics vary across seasons.

In addition to temporal dynamics, the relationship between urban green spaces and mental health may also exhibit nonlinear effects and spatial heterogeneity. Conventional attribution analysis typically relies on linear regression assumptions (29). However, recent studies have shown that the association between UGS and emotional well-being is often nonlinear and spatially heterogeneous (30), likely reflecting differences in urban form, population structure, and environmental context (31). Based on this, machine learning (ML) methods have attracted attention due to their proficiency in handling nonlinearities in large-scale data; yet they are usually implemented as global models with insufficient spatial interpretability (32). To address this limitation, geographically weighted ML techniques have gradually emerged. Among them, geographically weighted random forests (GWRF) construct an independent local random forest regression model at each spatial location and enhance interpretability through feature importance (33). By conducting nonlinear modeling while fully considering spatial heterogeneity, these methods can deepen our understanding of the complex associations within the model and have been applied in fields such as urban planning (34), medicine (33), and transportation analysis (30). This makes GWRF particularly suitable for this study, which aims to examine seasonally varying UGS–emotion associations that may differ across communities in magnitude (35, 36).

Overall, three key gaps remain in the existing studies. First, many studies focus on limited UGS indicators and do not jointly consider quantity, visibility, quality, and landscape-related characteristics. Second, seasonal dynamics are often overlooked, despite the fact that both green space conditions and residents’ emotional experiences may vary substantially across the year. Third, insufficient attention has been paid to the nonlinear and spatially heterogeneous nature of these associations. Addressing these gaps is essential for developing a more comprehensive understanding of how UGS relate to negative emotions.

This study aims to examine the seasonal associations between multidimensional UGS characteristics and negative emotions, and to identify the relative importance of different indicators across seasons. To this end, we leverage social media data to quantify seasonal patterns of negative emotions across different time periods. Additionally, we introduce the GWRF method to reveal the nonlinear relationships and spatial heterogeneity in the associations between UGS indicators and negative emotions across seasons.

## 2 Study area and data

### 2.1 Study area

**Figure 1:**
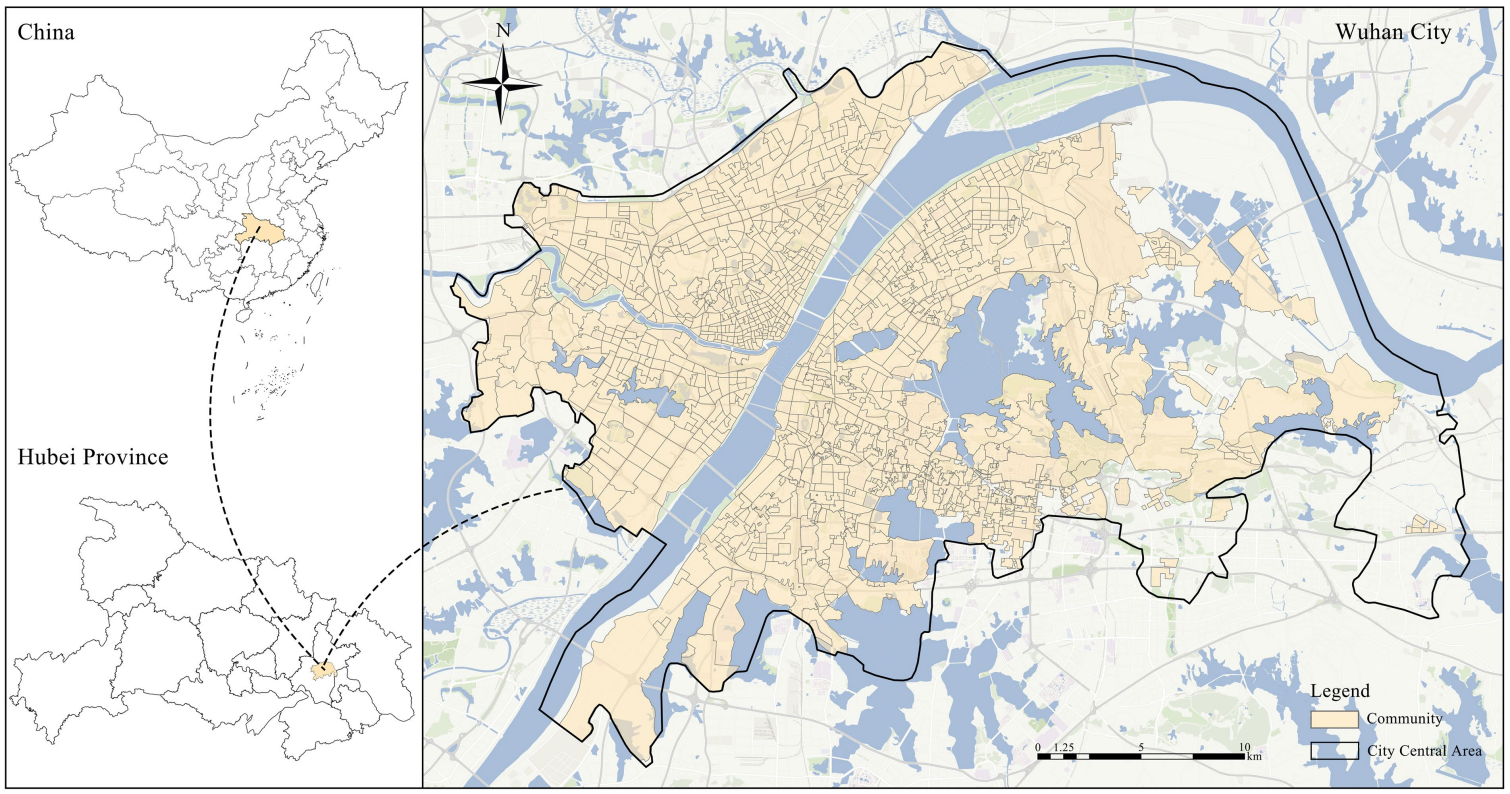
Location of the study area.

Wuhan was selected as the study area for this research. Located in central China, it is a major metropolis in the middle reaches of the Yangtze River, with a total jurisdictional area of 8,569.19 km^2^ and a permanent resident population of approximately 13.81 million. Wuhan has a subtropical monsoon climate, where both climatic conditions and green space landscape characteristics exhibit distinct seasonal variations. Meanwhile, the city contains numerous lakes, including East Lake, and is therefore hailed as the “City of a Hundred Lakes” (37). Its abundant natural resources and pronounced seasonal environmental variation provide a suitable research setting for exploring the relationship between UGS and mental health-related emotional outcomes. Over the past few decades, Wuhan has undergone rapid industrialization and urbanization, with a continuous increase in urban construction intensity. Consequently, optimizing green space allocation and maximizing its health benefits have become an important requirement for improving the level of urban public health (30). Taking Wuhan as a representative case city, this study examines the seasonal associations between multidimensional UGS characteristics and negative emotions, thereby providing a reference for healthy city construction and green space planning. We selected 2022 as the observation period for two reasons. First, it provided a complete annual window for comparing the associations between UGS and negative emotions across four seasons. Second, 2022 represented a late-pandemic context in which daily activities were gradually resuming, while epidemic-control measures still varied across time and space in Wuhan. Such contextual variation could affect residents’ opportunities for outdoor activities and green space use, as well as their negative emotional expressions on social media. To reduce this potential source of confounding, we further constructed a lockdown intensity variable and included it as a control variable in all subsequent analyses (Figure 1).

### 2.2 Influencing factors

This study explores the associations between multidimensional UGS characteristics and community-level negative emotions from three dimensions: green space quantity, green space quality, and landscape pattern. Based on a systematic review of relevant literature and considerations of data availability, we ultimately selected a set of common influencing factors in mental health research (Table 1).

**Table 1:** Description of influencing factors.

Categories	Factors	Description	Source
Green space quantity	NDVI	Normalized difference vegetation index for each unit.	RESDC (https://www.resdc.cn/)
	GVI	Green view index for each unit.	Baidu map (https://map.baidu.com/)
Green space quality	Outdoor public space accessibility	Walkability index for accessibility from community to outdoor public spaces.	Baidu map (https://map.baidu.com/)
	Green space accessibility	Walkability index from the community to the green space.	Baidu map (https://map.baidu.com/)
	Green space openness	Openness index of green spaces.	Baidu map (https://map.baidu.com/)
	Walkability	Walkability index for community streetscapes.	Baidu map (https://map.baidu.com/)
Landscape pattern	Largest patch index (LPI)	This index measures the proportion of the largest patch in a landscape, indicating the dominance of a single patch within the landscape.	FRAGSTATS *v.4.2*
	Patch density (PD)	The index quantifies landscape fragmentation by counting the number of patches (landscape elements) per unit area.	FRAGSTATS *v.4.2*
	Shannon’s diversity index (SHDI)	This index is used to assess landscape diversity, encompassing both species richness and evenness.	FRAGSTATS *v.4.2*
	Edge density (ED)	The length of edges per unit area is a critical indicator for measuring the degree of landscape fragmentation.	FRAGSTATS *v.4.2*
Control factors	Residential density	The ratio of residential area to total area within each unit.	Amap (https://www.amap.com/)
	Proportion of females	Percentage of female population in each unit.	WorldPop (https://hub.worldpop.org/)
	Night light index	Intensity of nighttime artificial light within each unit.	National Earth System Science Data Center (https://www.geodata.cn/data/)
	Lockdown intensity	The ratio of the duration during which each community was under lockdown to the total study period.	WMHC (https://wjw.wuhan.gov.cn/sy/)

NDVI characterizes the quantity of greenery in a community, with higher NDVI values often associated with fewer negative emotions (38). GVI reflects the visibility of green spaces from a pedestrian perspective, and abundant visible green spaces contribute to promoting mental health (39). Outdoor public space accessibility and green space accessibility indicate the convenience for residents to walk to outdoor public spaces and green spaces, respectively (40). Green space openness influences the frequency of social participation and interactions, and it is recognized as a key factor in improving mental health (41). Walkability reflects the level of support provided by the street environment for walking, which can improve mental health by facilitating daily physical activity (42).

In terms of landscape patterns, a higher Largest Patch Index (LPI) can provide more stable and continuous restorative spaces, thereby alleviating negative emotions and promoting mental health (17, 43). Conversely, higher Patch Density (PD) and Edge Density (ED) reduce the sense of safety and willingness to use green spaces, which in turn decreases usage frequency, weakens the emotional restorative effect, and is detrimental to mental health (43). The Shannon’s Diversity Index (SHDI) further enhances mental health benefits by increasing the diversity of leisure and social scenarios (44).

Higher residential density is often associated with poorer mental health status (45). The female group is more active on social media, and spatial differences in their distribution may affect the mental health characteristics depicted using social media data (58, 59). Night light index is highly correlated with the level of economic activity, and residents’ mental health is usually closely linked to the level of regional economic development (38).

In addition, because epidemic-control measures in Wuhan still varied across communities and time periods in 2022, lockdown intensity was introduced as an additional control factor. This variable was used to account for the potential influence of mobility restrictions on both residents’ actual opportunities to access green spaces and their negative emotional expressions (16).

## 3 Methodology

### 3.1 Research framework

**Figure 2:**
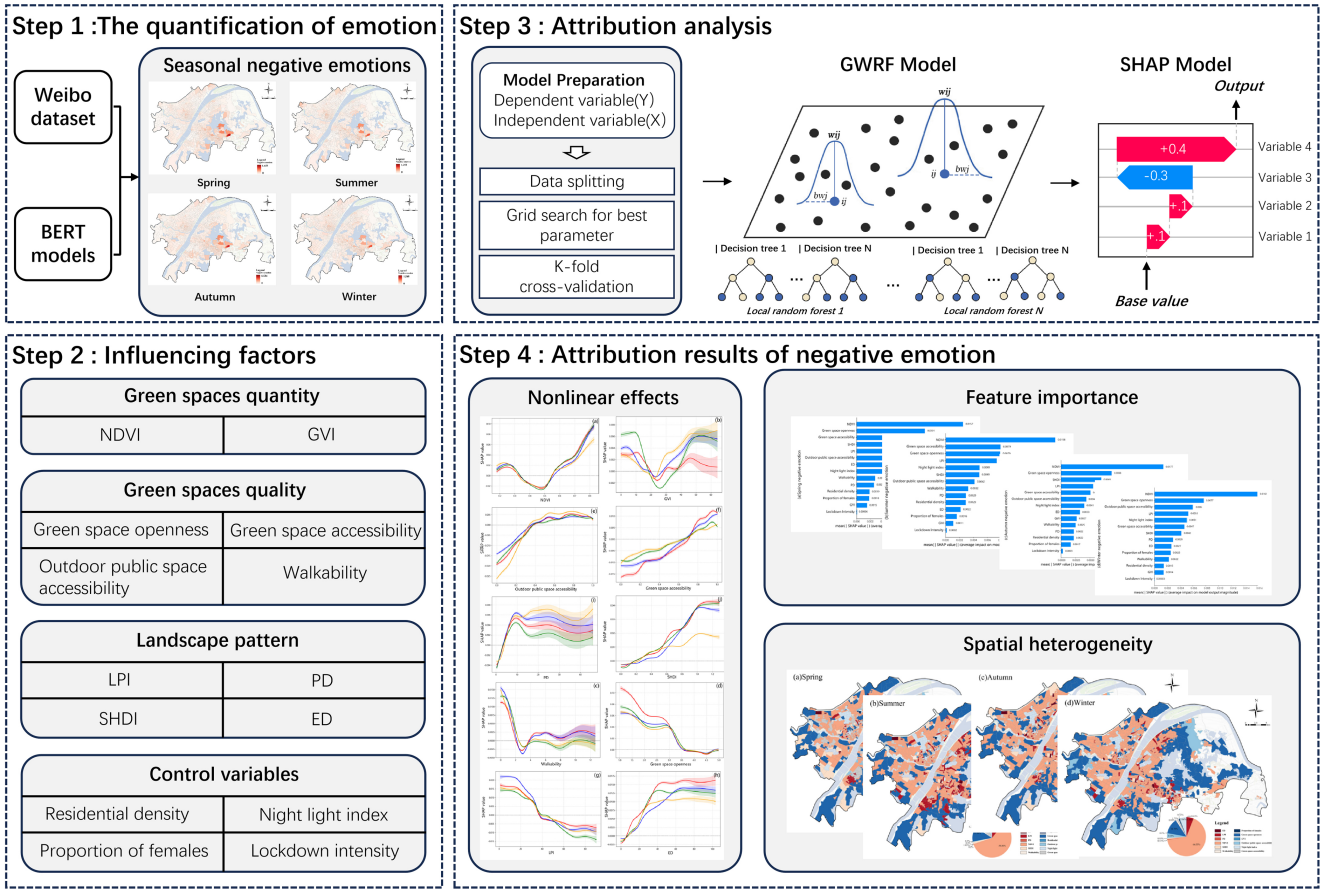
Research framework.

As illustrated in Figure 2, this study consists of four steps. First, information was extracted from the Weibo dataset based on a fine-tuned BERT model to obtain the spatial distribution of negative emotions. Second, multidimensional UGS indicators and control variables spanning three dimensions were selected for subsequent attribution analysis. Third, the GWRF and SHAP models were introduced to conduct attribution analysis of negative emotions. Finally, the results were interpreted in terms of feature importance, nonlinear effects, and spatial heterogeneity.

### 3.2 Calculation of negative emotions

As of September 2021, Sina Weibo had 511 million monthly active users, providing an abundant empirical basis for research on public emotions (10). Similar to Twitter (X), Sina Weibo allows users to voluntarily share location information when posting, thereby enabling geotag-based social perception research (46). Using the Sina Weibo Open Application Programming Interface (API), this study collected Weibo check-in data within the boundary of Wuhan City from January 1 to December 31, 2022. After deduplication processing, a total of 158,108 valid posts were obtained. Each post contains user ID, text content, posting time, posting location, and other relevant information.

To identify negative posts, the pretrained bert-base-chinese model was selected as the basic framework to build a sentiment classifier. Built on the Transformer architecture and trained via unsupervised pretraining, this model provides strong general-purpose representations for Chinese text (47). However, since the original pretrained model is not specifically optimized for sentiment classification tasks, we further fine-tuned it downstream to improve its task adaptability (48).

The fine-tuning process comprised three steps. First, we randomly sampled posts from the check-in dataset and manually labeled them into three classes (positive, neutral, and negative) with 1,000 posts per class. Second, we split the labeled data into training, validation, and test sets based on a 6:2:2 ratio. Third, we fine-tuned model parameters on the training set, selected optimal hyperparameters and model checkpoints using the validation set, and evaluated final performance on the test set. The model performance was assessed using Accuracy, Precision, Recall, and F1-score. The optimal model was then applied to the remaining unlabeled posts to perform sentiment classification. For subsequent analyses, we retained only posts classified as negative and used them to construct a community-level indicator of negative emotional expression. To reduce the potential bias introduced by differences in community population size, the resulting indicator was further standardized by the permanent resident population of each community before empirical modeling.

### 3.3 Kernel density estimation

Weibo check-in posts provide geotagged observations as discrete point records, whereas negative emotions are more appropriately conceptualized as spatially continuous. We therefore apply kernel density estimation (KDE) to transform point observations into a smooth spatial intensity surface. KDE is a nonparametric technique that assigns each observation a kernel-based weight and aggregates these contributions across space, yielding a continuous density field. Areas with higher estimated densities are interpreted as locations where negative emotional expressions are more concentrated. KDE has been widely used in emotion and sentiment research and was used here to characterize the spatial distribution of negative emotional expression (49). The formula is expressed as follows:

$$\hat{f}(s)=\sum_{i=1}^{n}\frac{1}{nh^{2}}K(\frac{s-s_{i}}{h})$$ (1)

In Equation 1, $\hat{f}(s)\;$ denotes the kernel density estimate at location s ; n is the number of negative emotion points; h represents the bandwidth; K is the kernel function; and $s-s_{i}$ denotes the distance between location s and observation $s_{i}$ . We implemented KDE in ArcGIS Pro to estimate the spatial intensity surface of negative emotions in Wuhan for the full year. The KDE values were then normalized by the permanent resident population of each community to generate the final negative emotion indicator used in subsequent analyses.

### 3.4 Calculation of urban green space indicators

#### 3.4.1 Green space quantity

NDVI was computed from multispectral imagery using surface reflectance in the near-infrared (NIR) and red bands as:

$$\text{NDVI}=\frac{\mathrm{NIR}-\mathrm{Red}}{\mathrm{NIR}+\mathrm{Red}}$$ (2)

In Equation 2, NDVI ranges from -1 to 1. Negative values typically correspond to clouds, water, or snow, whereas values near zero generally indicate rock or bare soil. Positive values represent green vegetation, and higher NDVI indicates greater vegetation density, implying a higher likelihood that residents are exposed to green spaces (50). We calculated NDVI using GF-2 satellite imagery acquired in 2022.

Street-view images were obtained from the Baidu Maps Street View platform. Sampling points were placed at 100 m intervals along the public road network within the study area, and four images representing different viewing directions were collected at each point. We applied the FCN-8 s semantic segmentation model to classify street-view imagery, identify vegetation elements (eg., trees and grass), and compute GVI as the proportion of vegetation pixels to total pixels in each image (58). Community-level GVI was aggregated in two steps: (1) averaging GVI across the four directional images at each sampling point; and (2) averaging these point-level values across all sampling points within each community to obtain a community-level street-view greenness indicator (56).

#### 3.4.2 Green space quality

UGS quality was operationalized across four dimensions: outdoor public space accessibility, green space accessibility, green space openness, and walkability. For outdoor public space accessibility and green space accessibility, we constructed a 1 km network-based walking catchment centered on each community, reflecting a plausible distance residents are willing to walk in daily life to access nearby resources. Within this catchment, we used Baidu Maps point-of-interest (POI) data with geographic coordinates to quantify outdoor public space accessibility and green space accessibility based on the availability of corresponding destinations within the network-based walking catchment, represented by the number of outdoor public spaces and green spaces, respectively (51).

For UGS openness and walkability, we deployed street-view sampling points along the road network and retrieved multi-directional street-view images at each point. We applied the FCN-8 s semantic segmentation model to identify key scene elements (eg., trees, grass, roads, sidewalks, and buildings). Using a set of manually scored samples, we trained regression models to predict green space openness and walkability for all sampling points. Predicted values were then averaged across viewing directions at each sampling location and subsequently aggregated to the community level by averaging across all sampling points, yielding community-level indicators of green space openness and walkability (10).

#### 3.4.3 Landscape pattern

Following the FRAGSTATS User’s Guide and recent methodological syntheses, we selected four representative landscape metrics: LPI, ED, SHDI, and PD. These metrics were computed in FRAGSTATS 4.2 using land-use raster data.

#### 3.4.4 Attribution analysis

##### 3.4.4.1 GWRF model

As a hybrid method combining geographically weighted modeling and ensemble decision trees, GWRF can simultaneously account for spatial heterogeneity and complex nonlinear relationships between predictors and outcomes (52). Its core idea is as follows: for each sample point at a spatial location (ui,vi) , a local random forest submodel is built within its neighborhood, and local calibration is achieved through spatial weights, thereby capturing the differentiated effects of the same factors across locations. The GWRF model is calculated as follows:

$$y_{i}=a(\mu_{i},v_{i})x_{i}+e$$ (3)

In Equation 3, where $y_{i}$ is the negative emotion of the i -th community; a(ui,vi) denotes the random forest mapping relationship locally calibrated at location a(ui,vi) and e is the residual term. For each observation, the GWRF model builds and runs the corresponding RF submodel within its neighborhood (or kernel function) range; the maximum distance between an observation and the sample points within its neighborhood is defined as the bandwidth. Two types of neighborhoods are commonly used: “adaptive” and “fixed.” The former defines the neighborhood using the 𝑛 nearest community points, whereas the latter uses a circular window with the bandwidth as the radius. Considering that community points are unevenly distributed in space and that community-level negative emotions exhibit significant spatial heterogeneity, this study adopts an adaptive neighborhood so that the window size can automatically adjust to local variability, thereby more effectively capturing spatial non-stationarity and improving predictive stability. Compared with conventional RF, GWRF is better suited to this study because it allows the relationships identified by the model to vary across space rather than assuming a single global pattern. Compared with traditional GWR, it is more flexible in capturing nonlinear associations (35, 36).

In addition, we introduced traditional random forest (RF) and geographically weighted regression (GWR) as benchmark models. All models were evaluated using $R^{2}$ , mean absolute error (MAE), and root mean square error (RMSE). The formulas are as follows:

$$R^{2}=1-\frac{\sum_{i=1}^{n}{\left(y_{i}-{\hat{y}}_{i}\right)}^{2}}{\sum_{i=1}^{n}{\left(y_{i}-\bar{y}\right)}^{2}}\;\;$$ (4)

$$\begin{array}{c} \mathrm{MAE}=\frac{1}{n}\sum_{i=1}^{n}|y_{i}-{\hat{y}}_{i}| \end{array}$$ (5)

$$\begin{array}{c} \text{RMSE}=\sqrt{\frac{1}{n}\sum_{i=1}^{n}{\left(y_{i}-\hat{y_{i}}\right)}^{2}}\; \end{array}$$ (6)

In Equations 4–6, where $y_{i}$ is the observed emotion value for the i -th community; $\hat{y_{i}}$ is the predicted emotion value for the i -th community; $\bar{y}$ is the mean observed emotion value; and *n* is the number of communities.

##### 3.4.4.2 SHAP model

Most ML models are often criticized for their limited interpretability, as their predictive mechanisms are not readily transparent. SHapley Additive exPlanations (SHAP) provides a unified framework for interpreting ML models by computing Shapley values for each factor, which quantify that factor’s marginal contribution to the model prediction. Based on this, we can identify the factors that contribute most to model predictions and distinguish relatively influential variables from those with comparatively smaller contributions. The Shapley value ( ϕ ) is defined as:

$$\begin{array}{c} \phi_{i}=\sum_{S\subseteq N\{i\}}\frac{|S|!(n-|S|-1)!}{n!}[v(S\cup i)-v(S)]\; \end{array}\;\;$$ (7)

In Equation 7, $\phi_{i}$ denotes the Shapley value for factors i , quantifying its contribution (importance) to the model prediction. Let N be the set of all factors with *∣N∣ = n*. For any subset S ⊆ N∖{i}, v(S) denotes the model output when only factors in S are included, and v(S∪{i}) denotes the model output after adding factors i to S .

## 4 Results

### 4.1 Spatiotemporal distribution patterns of negative emotions

#### 4.1.1 Temporal distribution

**Figure 3:**
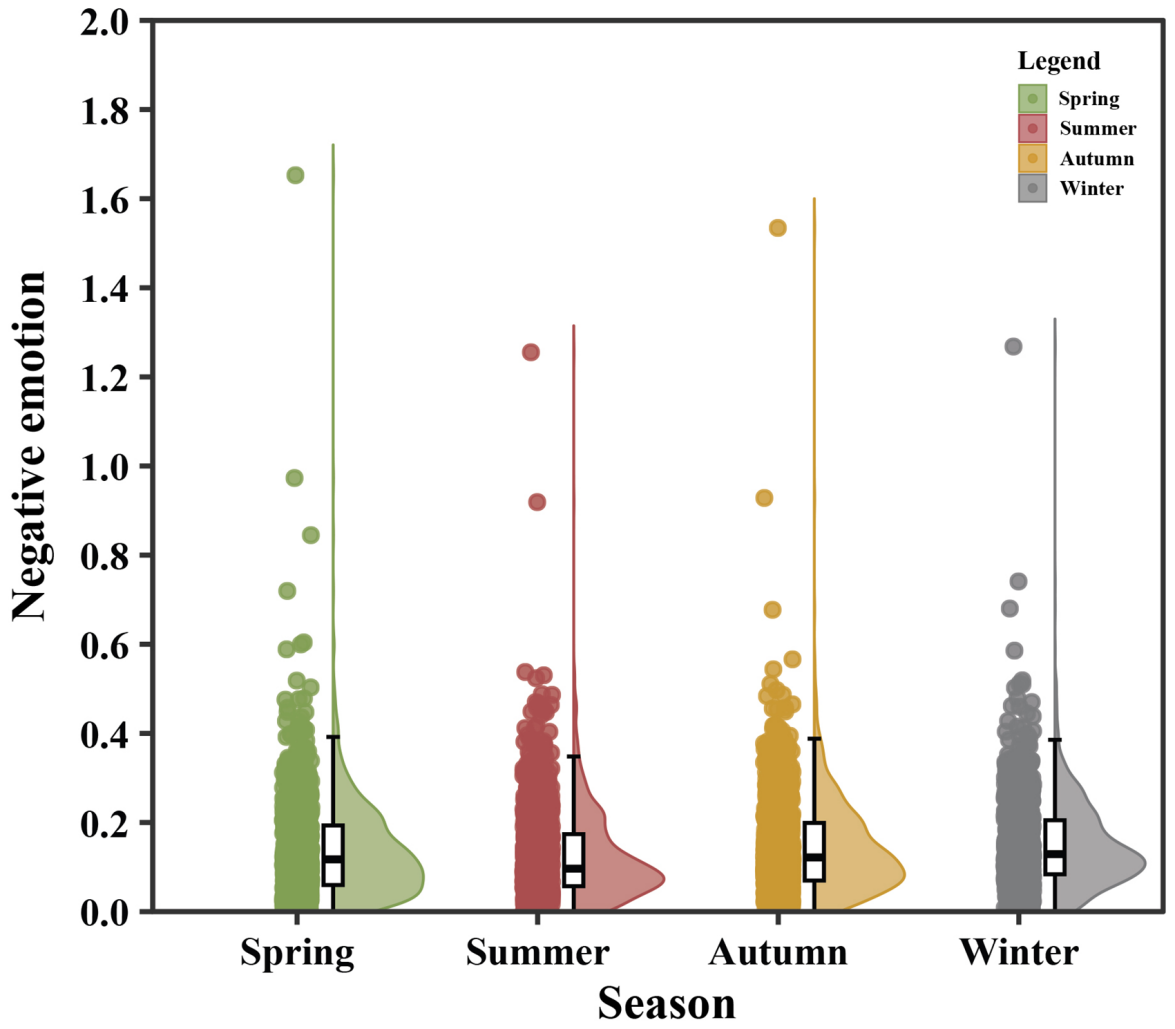
Population-standardized negative emotion indicator across four seasons.

According to the meteorological division of seasons, spring covers March–May, summer June–August, autumn September–November, and winter December–February of the following year (53). Figure 3 illustrates the seasonal variations in the population-standardized negative emotion indicator. Specifically, the average negative emotion index was 0.1386 in spring, reached its lowest value of 0.1244 in summer, increased to 0.1436 in autumn, and peaked at 0.1508 in winter, showing an overall trend of first decreasing and then increasing.

#### 4.1.2 Overall spatial distribution

**Figure 4:**
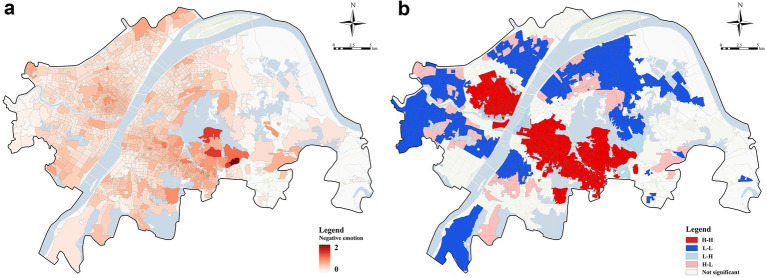
**(a)** Spatial distribution of negative emotion in the community; **(b)** Spatial clustering of negative emotion based on the local Moran’s I.

Figure 4a illustrates the spatial distribution of community-level negative emotions. Negative emotions exhibit a pronounced core–periphery structure, with areas of higher negative emotions predominantly concentrated in the central region and along the southern periphery. In contrast, most other regions show generally lower levels of negative emotions. To further explore the spatial pattern, we examined the global spatial autocorrelation of community-level negative emotions using the Global Moran’s I. The Moran’s I value for the negative emotion indicator was 0.439 (*p* < 0.0001), indicating statistically significant positive spatial autocorrelation at the annual scale.

Figure 4b presents the Local Moran’s I (LISA) results for negative emotions. High–high (HH) clusters are primarily concentrated in the central and southern parts of Wuhan, while low–low (LL) clusters are focused in the eastern and western regions. High–low (HL) and low–high (LH) outliers, representing areas with relatively high or low negative emotions, are distributed in the transitional zones between these two types of clusters.

#### 4.1.3 Seasonal spatial distribution

**Figure 5:**
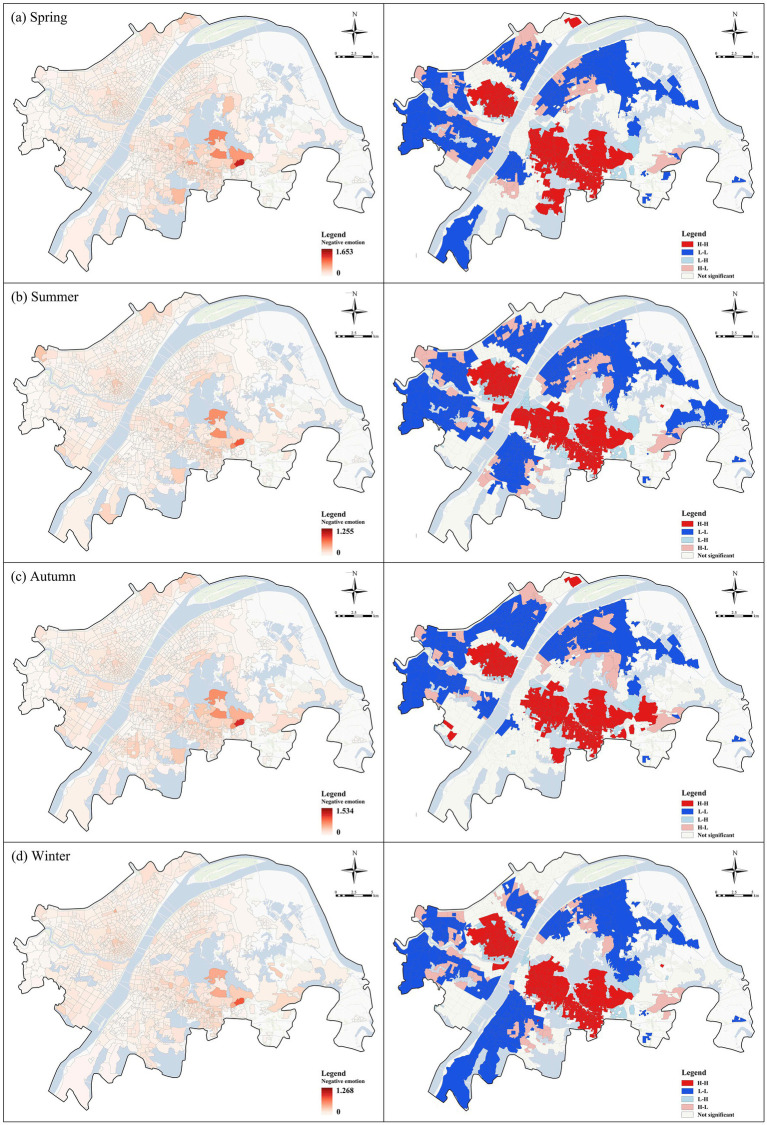
Spatial distribution of negative emotion across four seasons in the community.

Figure 5 illustrates the seasonal differences in the spatial distribution of negative emotions. Overall, the spatial patterns are broadly consistent across seasons, although the extent and intensity of high-value areas vary. High-value clusters of negative emotions are predominantly concentrated in the urban core and southern regions, while the urban periphery is mostly characterized by low values. Nevertheless, the extent and intensity of high-value clustering vary somewhat by season. In spring, negative emotions exhibit significant clustering in the southern and central regions (Figure 5a). In summer, the spatial extent of high-value areas contracts, with negative emotions concentrated mainly in the central region (Figure 5b). In autumn, negative emotions intensify again in the southern region (Figure 5c). In winter, pronounced high-value clustering emerges (Figure 5d). Season-specific spatial autocorrelation analysis further confirmed that positive spatial clustering remained statistically significant in all four seasons, although the magnitude and local clustering extent varied seasonally.

### 4.2 Global results

#### 4.2.1 Model comparison

Table 2 summarizes the performance of different models. Across both the full-year and seasonal scenarios, the RF model exhibited relatively low goodness of fit and large prediction errors. The GWR model showed a significant improvement in R^2^ values after incorporating local spatial regression. Compared with the conventional models, the GWRF model performed superiorly in terms of goodness of fit, achieving higher R^2^ values as well as lower MAE and RMSE than the other models. When comparing the model fit across the four seasons, GWRF performed relatively better in autumn and winter, but slightly poorer in summer. Overall, the GWRF model consistently outperformed the other models across all seasonal scenarios, demonstrating better robustness and overall performance. These findings suggest that the GWRF model can more effectively explain the association between UGS characteristics and negative emotions.

**Table 2:** Performance parameters of RF, GWR, and GWRF models.

Seasons	Models	R^2^	MAE	RMSE
Annual negative emotion	RF	0.169	0.146	0.180
	GWR	0.420	0.119	0.154
	GWRF	0.746	0.071	0.102
Spring negative emotion (Mar–May)	RF	0.148	0.057	0.082
	GWR	0.393	0.064	0.091
	GWRF	0.749	0.039	0.059
Summer negative emotion (June–August)	RF	0.151	0.067	0.089
	GWR	0.351	0.061	0.085
	GWRF	0.703	0.038	0.058
Autumn negative emotion (September–November)	RF	0.179	0.069	0.086
	GWR	0.391	0.062	0.086
	GWRF	0.756	0.036	0.054
Winter negative emotion (December–February)	RF	0.147	0.069	0.091
	GWR	0.365	0.059	0.081
	GWRF	0.715	0.036	0.054

#### 4.2.2 Global relative importance

**Figure 6:**
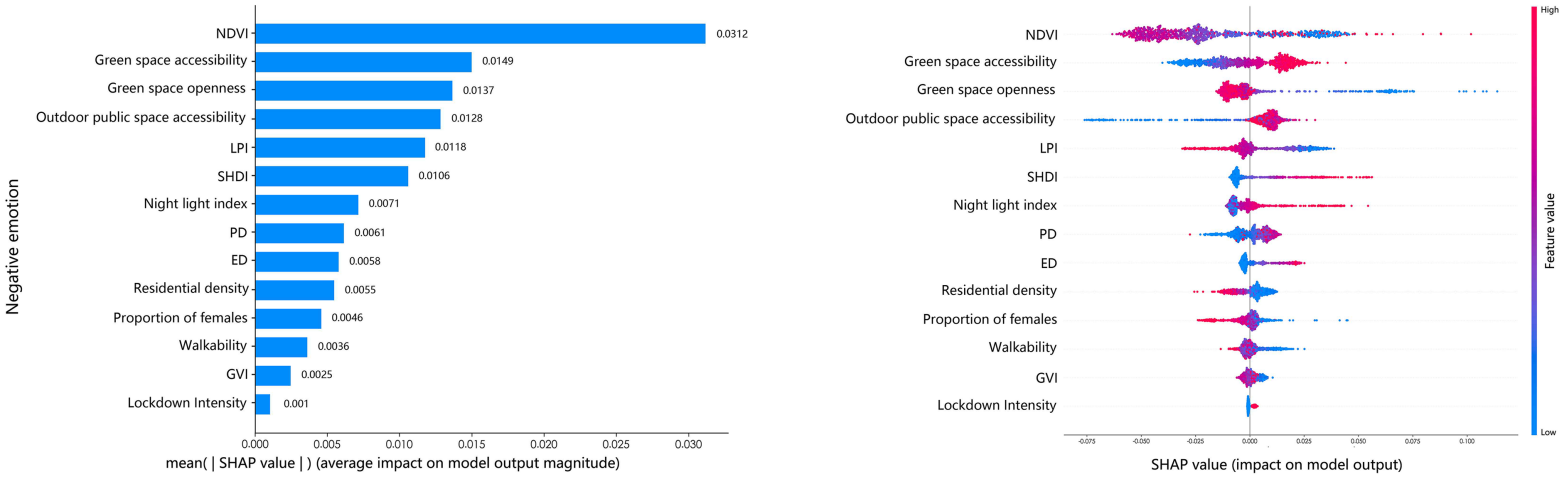
Relative importance of urban green space factors on annual negative emotion.

Figure 6 shows the ranking of influencing factors based on their global average SHAP values with respect to negative emotions over the full year. The results indicate that NDVI, green space accessibility, and green space openness make the largest contributions to model predictions of negative emotions. Second, outdoor public space accessibility and LPI are also closely associated with negative emotions. In addition, SHDI ranks in an intermediate position. However, GVI exhibits a relatively low ranking in terms of global importance.

**Figure 7:**
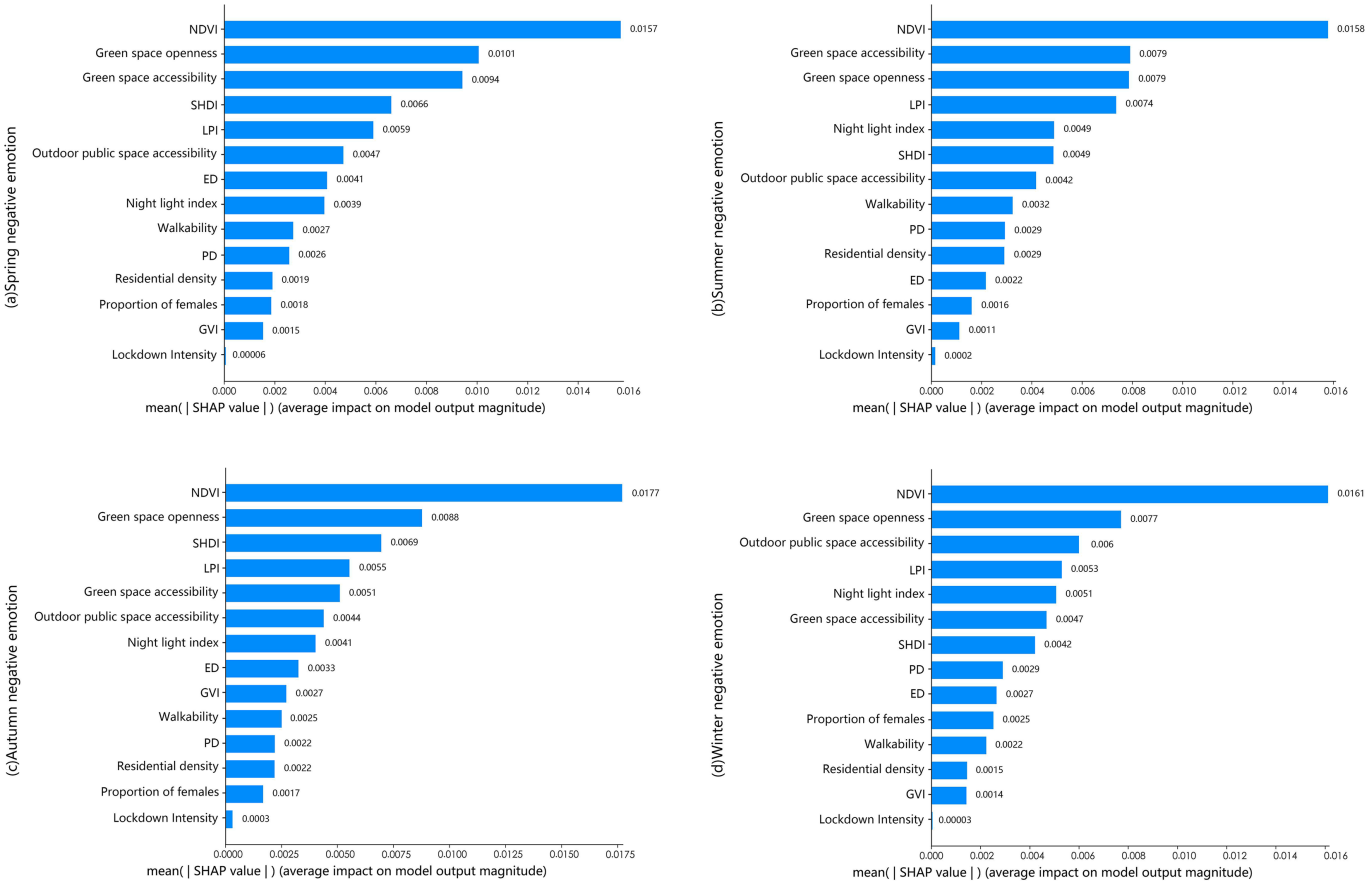
Relative importance of urban green space factors on public negative emotions in **(a)** spring, **(b)** summer, **(c)** autumn, and **(d)** winter.

Figure 7 illustrates the seasonal variations in the relative importance of influencing factors with respect to negative emotions. Overall, NDVI, green space accessibility, green space openness, and LPI consistently maintain high importance across all four seasons. Among these factors, NDVI ranks first in every season, highlighting that its influence on negative emotions is stronger than that of other factors. In contrast, the lockdown intensity and GVI remain consistently low in importance across all seasons. Notably, pronounced seasonal variations in the importance of several indicators were observed. For instance, green space accessibility ranks third in spring (Figure 7a), jumps to second in summer (Figure 7b), and declined to sixth in winter (Figure 7d).

#### 4.2.3 Nonlinear effects

**Figure 8:**
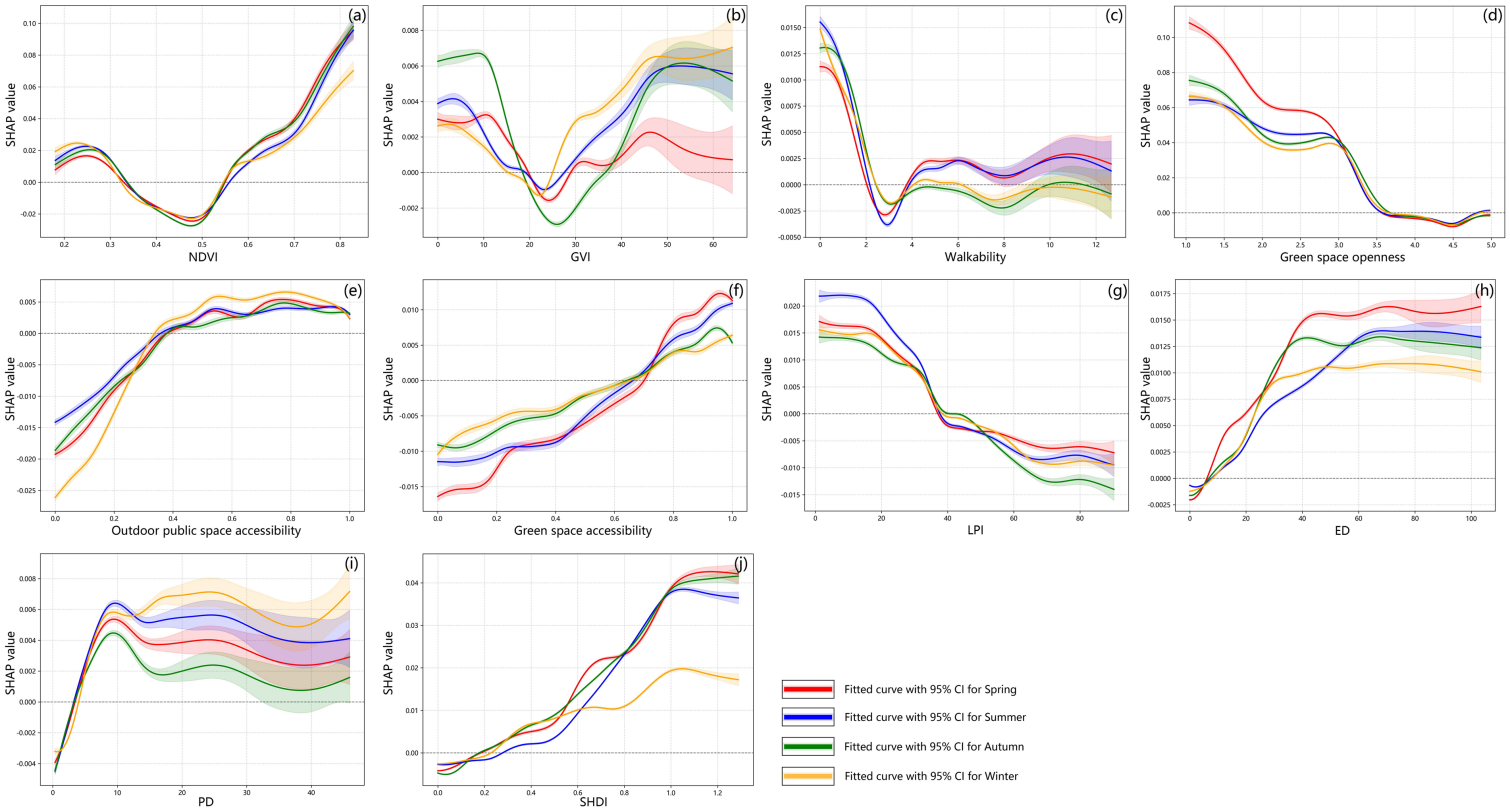
PDPs of urban green space factors and negative emotions across four seasons.

Figure 8 presents seasonal partial dependence plots (PDPs) illustrating the relationships between each influencing factor and negative emotions across the four seasons. Specifically, the green, red, blue, and yellow curves represent the season-specific fitted curves and their corresponding 95% confidence intervals, respectively. Overall, all features exhibit pronounced nonlinear and threshold characteristics. However, these characteristics show typical seasonal differences in several indicators.

##### 4.2.3.1 Green space quantity

NDVI and GVI exhibit broadly similar overall trends in their effects on negative emotions across different seasons, but there are significant seasonal differences in their effect magnitudes. For NDVI (Figure 8a), the effect shifts from negative to positive in all four seasons, with a consistent turning threshold around 0.5; notably, this shift is most pronounced in spring and least significant in winter. For GVI (Figure 8b), the primary threshold is approximately 20%. The negative effect of GVI attenuates after exceeding 20% but remains positive overall.

##### 4.2.3.2 Green space quality

Outdoor public space accessibility (Figure 8e), green space accessibility (Figure 8f), and walkability (Figure 8c) all exhibit an overall trend of transitioning from negative to positive effects across the four seasons, with turning thresholds of 0.4, 0.7, and 4, respectively. Notably, the magnitude of this transition is most pronounced in spring. Green space openness (Figure 8d) shows a distinct nonlinear threshold effect, with its effect gradually shifting from positive to negative and then turning positive again across all seasons. Among these seasons, the positive effect is strongest in spring and relatively weaker in winter.

##### 4.2.3.3 Landscape pattern

LPI (Figure 8g) exhibit a trend of transitioning from positive to negative effects across the four seasons. The magnitude of this transition is most pronounced in summer and weakest in spring, with a turning threshold around 40%. In contrast, ED (Figure 8h), PD (Figure 8i), and SHDI (Figure 8j) shows a transition from negative to positive effects, with a threshold of around 5, 4, and 0.2, respectively.

### 4.3 Local results

#### 4.3.1 Spatial heterogeneity of dominant influencing factors

**Figure 9:**
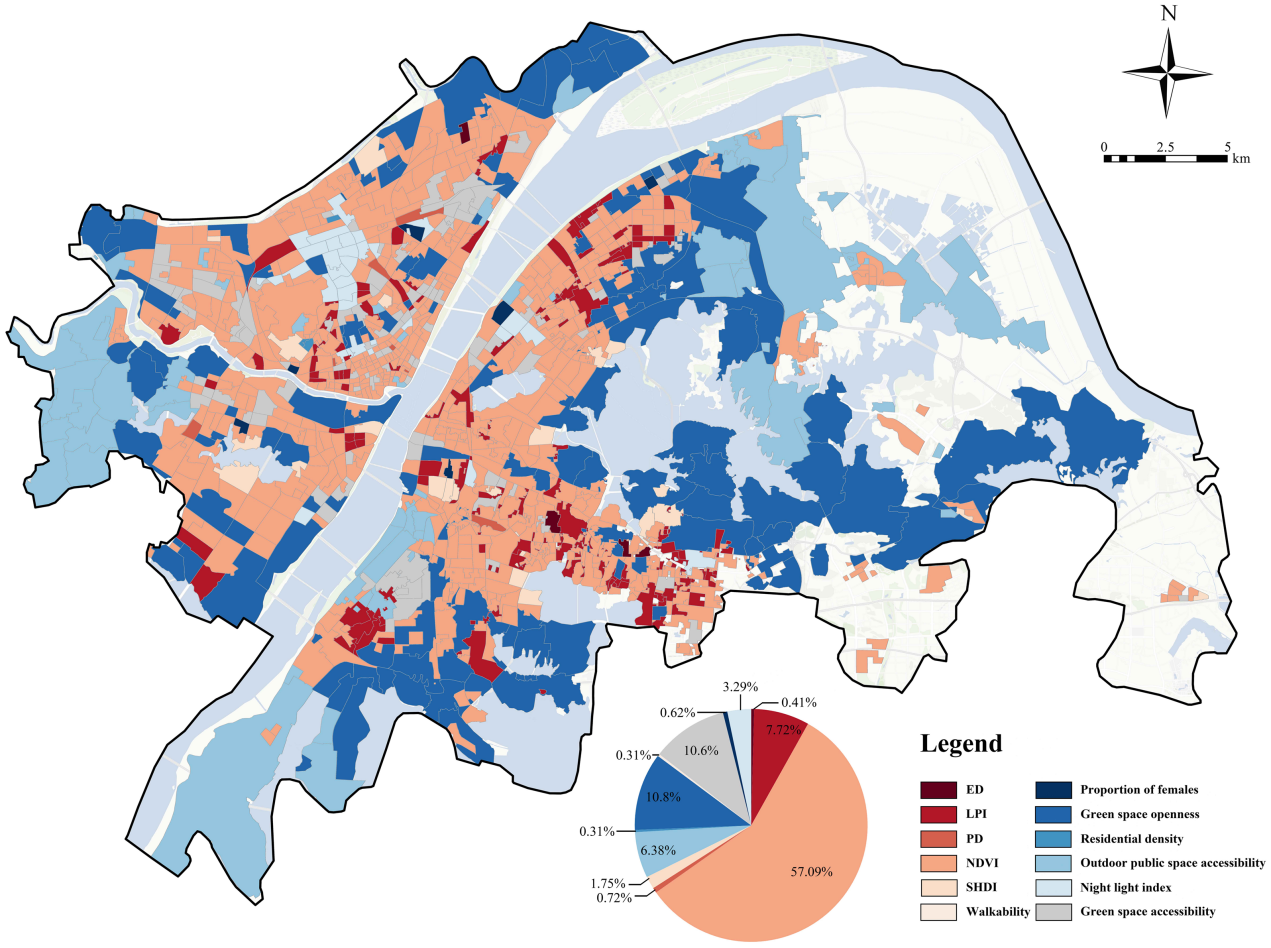
Spatial distribution of primary factors influencing annual negative emotion.

Based on the results presented in Figures 6, 7, we further identified the dominant factors influencing negative emotions at the local scale, focusing on the NDVI, outdoor public space accessibility, green space accessibility, green space openness, and LPI (Figure 9). Our findings revealed significant spatial heterogeneity in these dominant factors. NDVI emerged as the most prominent locally dominant factor, accounting for 57.09% of communities in which it made the largest contribution to model predictions of negative emotions. Additionally, the green space openness served as the dominant factor in 10.8% of communities, mainly showing clustered distributions in peripheral areas and around large ecological patches, where more open green environments may better differentiate local emotional outcomes. Green space accessibility and LPI were the dominant factors in 10.6 and 7.72% of communities, respectively. Green space accessibility mainly exhibited clustered distributions in communities with relatively abundant nearby green resources, whereas LPI showed more localized clustered patterns, particularly in parts of the central and southern urban areas. In contrast, residential density and walkability each accounted for less than 1% of communities as dominant factors.

**Figure 10:**
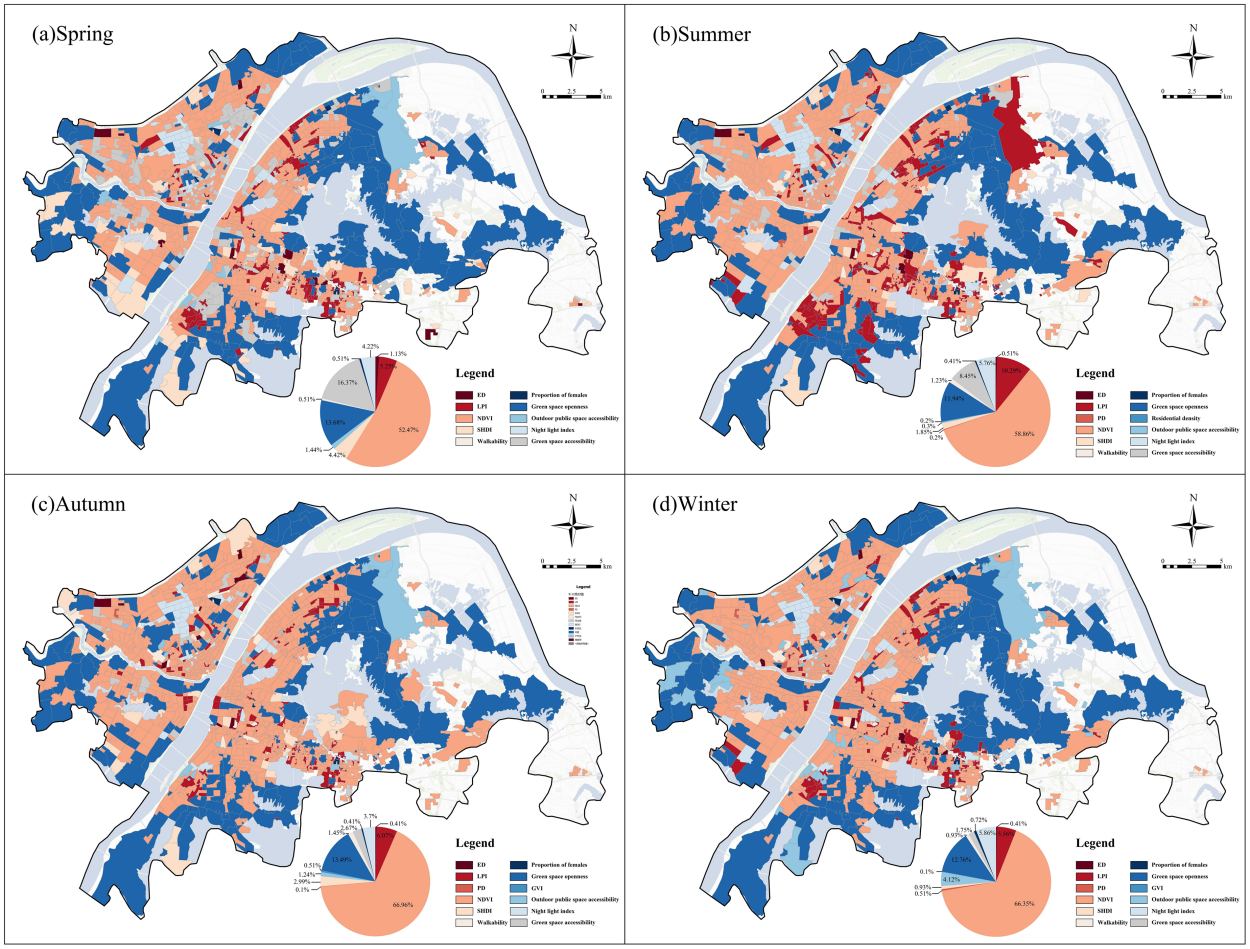
Spatial distribution of primary factors influencing negative emotions across four seasons. **(a)** Spring, **(b)** summer, **(c)** autumn, and **(d)** winter.

After identifying the dominant influencing factors of negative emotions across the full-year sample, we further analyzed the performance of these factors across different seasons. Significant seasonal differences were observed, although NDVI remained the most dominant factor during all seasons. In spring (Figure 10a), NDVI was the primary factor influencing negative emotions, accounting for 52.47% and concentrating mainly in urban fringe areas. Green space accessibility (16.37%) and green space openness (13.68%) exerted relatively strong influences, with their dominant communities distributed primarily in urban core areas. In summer (Figure 10b), the dominance of NDVI was further enhanced, with its proportion increasing to 58.86%, whereas the influences of green space openness (11.94%) decreased significantly. In autumn (Figure 10c), NDVI reached its highest proportion (66.96%), indicating that green space quantity showed the strongest association with residents’ negative emotions in autumn. In winter (Figure 10d), NDVI remained the most dominant factor (66.35%), although the influences of outdoor public space accessibility (4.12%) increased moderately.

#### 4.3.2 Spatiotemporal evolution of the key factors

We focused on the top three factors with the strongest influences on negative emotions, namely NDVI, green space openness, and green space accessibility. All three factors exhibited significant spatial heterogeneity in their associations with negative emotions, although their spatial patterns differed substantially. In general, NDVI and green space accessibility showed broadly similar spatial structures, with negative associations widely distributed in both the urban core and many peripheral communities, while positive associations were concentrated in several belt-like and fringe areas. By contrast, green space openness displayed a different pattern, with more positive local associations in peripheral communities and more negative local associations in parts of the central urban area.

##### 4.3.2.1 NDVI

**Figure 11:**
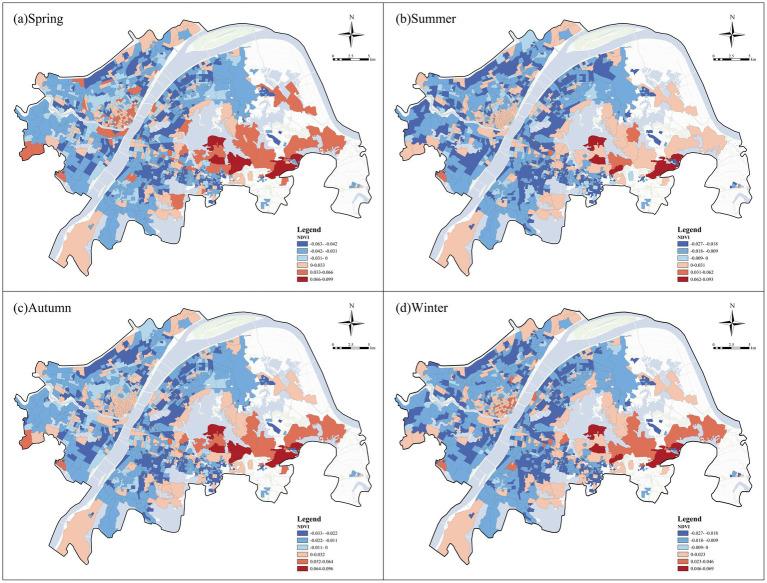
Spatial heterogeneity of NDVI on negative emotions. **(a)** Spring, **(b)** summer, **(c)** autumn, and **(d)** winter.

Figure 11 illustrates the spatial heterogeneity of the associations between NDVI and negative emotions across the four seasons. Overall, NDVI exhibited a broadly similar spatial pattern throughout the year. Negative associations were widely distributed in both the urban core and many peripheral communities, whereas positive associations were mainly concentrated in several belt-like and fringe areas, particularly in parts of the central-southern and eastern sections of the city. This pattern suggests that the relationship between NDVI and negative emotions varied substantially across urban contexts. From the perspective of seasonal variation, the broad spatial structure remained generally stable across the four seasons, although the magnitude and extent of associations changed to some degree. Positive associations were relatively more evident in spring, whereas negative associations became more extensive in some peripheral communities in autumn.

##### 4.3.2.2 Green space openness

**Figure 12:**
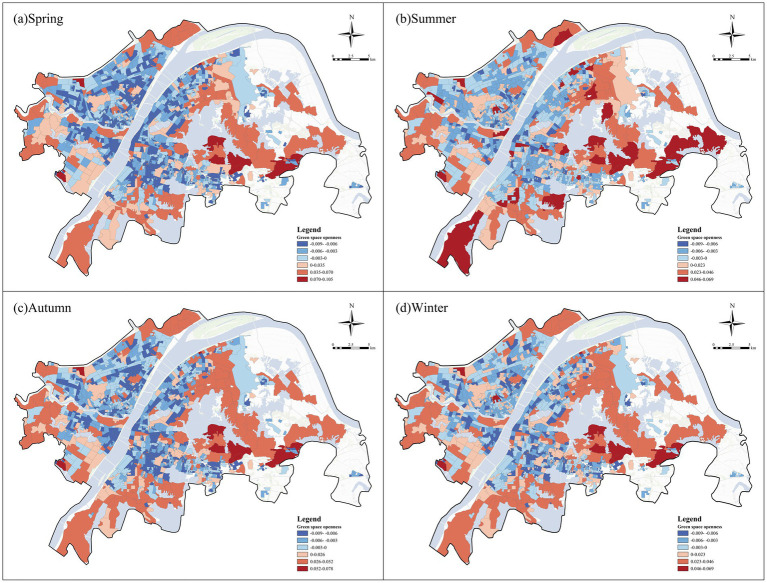
Spatial heterogeneity of green space openness on negative emotions. **(a)** Spring, **(b)** summer, **(c)** autumn, and **(d)** winter.

Figure 12 illustrates the spatial heterogeneity of the associations between green space openness and negative emotions across the four seasons. Green space openness showed positive associations in many peripheral communities and in parts of the eastern and southern urban areas, whereas more negative associations were concentrated in the urban core and several densely built-up inner-city zones. In terms of seasonal variation, the positive associations of green space openness were most pronounced in summer, when high-value positive areas expanded across peripheral communities and several outer urban zones. In autumn and winter, the overall positive pattern remained visible, although the magnitude of positive associations became relatively more moderate. Spring showed an intermediate pattern between these seasonal conditions.

##### 4.3.2.3 Green space accessibility

**Figure 13:**
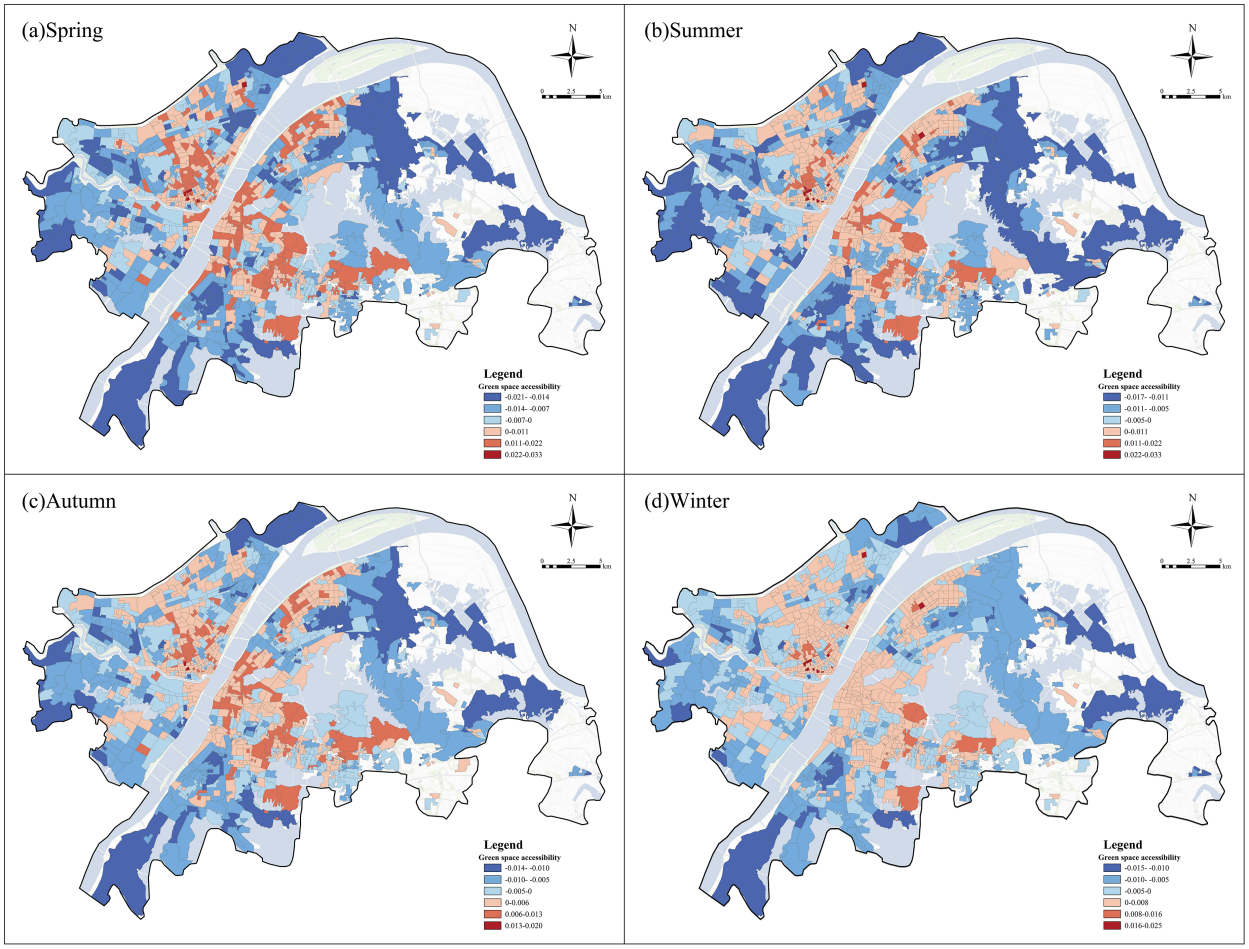
Spatial heterogeneity of green space accessibility on negative emotions. **(a)** Spring, **(b)** summer, **(c)** autumn, and **(d)** winter.

Figure 13 illustrates the spatial heterogeneity of the associations between green space accessibility and negative emotions across the four seasons. Across all four seasons, green space accessibility showed a broadly consistent spatial structure, with negative associations widely distributed in peripheral communities, especially in the eastern urban fringes, while positive associations were concentrated in several inner-city and belt-like areas. The positive associations of green space accessibility were more continuous in the central part of the city. From the perspective of seasonal variation, this overall spatial pattern remained generally similar across seasons, but the intensity and continuity of positive associations varied. Positive associations were more evident and spatially continuous in spring, whereas in winter they became relatively weaker in some central areas.

## 5 Discussion

### 5.1 Seasonal dynamics of the relationship between UGS and negative emotions

By explicitly incorporating seasonal dynamics, this study provides new insights into the associations between multidimensional UGS characteristics and negative emotions. Overall, the results indicate that these associations vary substantially across seasons in both magnitude and relative importance. Across the full year and the four seasonal models, NDVI consistently ranked first, while green space accessibility, green space openness, and LPI also remained important contributors in different seasons. In particular, green space accessibility showed stronger relative importance in spring than in autumn, whereas NDVI remained the most stable dominant factor throughout the year. These findings suggest that negative emotions are associated not only with baseline greenness, but also with quality and landscape patterns of urban green spaces. Consistent with previous studies, higher greenness is generally associated with more favorable emotional outcomes (50). However, our results further suggest that such associations are not temporally constant. Instead, the relative importance and modeled relationships of NDVI and other UGS indicators varied across seasons, implying that the relationship between green environments and emotional well-being is inherently dynamic. This seasonal variation may reflect the combined influence of vegetation phenology, climatic comfort, and residents’ outdoor activity patterns. For example, in spring and summer, when climatic conditions are relatively favorable, residents may have more opportunities to interact with nearby green environments, which could increase the importance of green space accessibility and openness (28). By contrast, in autumn and winter, opportunities for outdoor use may become more constrained, making baseline greenness a relatively more stable correlate of emotional outcomes (25).

The results also reveal pronounced nonlinear relationships and identifiable turning points between UGS characteristics and negative emotions. Most indicators exhibited relatively clear nonlinear patterns, and several turning points were broadly comparable across seasons. This suggests that, within the study context, there may be interpretable ranges at which the associations between UGS indicators and negative emotions begin to change direction or magnitude. For example, NDVI and GVI both showed evident turning points, while green space accessibility, green space openness, and several landscape metrics also displayed non-monotonic relationships. At the same time, the magnitude of these nonlinear relationships varied seasonally, with more pronounced changes in spring and relatively milder changes in winter. This seasonal contrast may reflect the fact that the perceptual and behavioral salience of green environments is more sensitive to environmental variation during climatically favorable periods (54). From a planning perspective, these findings suggest that attention should be paid not only to increasing or decreasing specific indicators, but also to identifying context-sensitive ranges in which their associations with emotional outcomes change more substantially.

Third, this study further reveals pronounced spatiotemporal heterogeneity in the associations between key UGS factors and negative emotions. Overall, NDVI, green space openness, and green space accessibility emerged as the three most important factors for examining spatial heterogeneity. Among them, NDVI and green space accessibility showed broadly similar spatial structures, with negative associations widely distributed in many urban core and peripheral communities, while positive associations appeared in several belt-like and fringe areas. By contrast, green space openness displayed a markedly different pattern, with more positive associations in peripheral communities and more negative associations in parts of the central urban area. This suggests that different dimensions of UGS may be associated with emotional outcomes through distinct spatial mechanisms. In seasonal terms, the broad spatial structures of these associations remained generally similar, but their magnitudes and spatial extents varied. NDVI showed relatively stable dominance throughout the year, while its negative associations became more extensive in some peripheral communities in autumn. Green space openness showed more pronounced positive associations in summer, whereas green space accessibility displayed more continuous positive associations in autumn. These differences imply that, even under a relatively stable baseline of urban greenness, the emotional relevance of different UGS characteristics may shift seasonally.

### 5.2 Policy implications for enhancing emotional health

First, the identified nonlinear relationships, turning points, and spatial heterogeneity suggest that UGS planning should not rely solely on the assumption that increasing a single indicator will necessarily lead to better emotional outcomes. Instead, both greenness-related and quality-related indicators showed non-monotonic relationships with negative emotions, implying that more refined and context-sensitive planning approaches may be needed. In this sense, the results provide a useful complement to conventional planning frameworks based primarily on linear assumptions.

The results showed that NDVI was consistently the primary factor explaining negative emotions throughout the year, indicating that a stable and sufficient quantity of green space was closely associated with alleviating negative emotions. Meanwhile, green space accessibility continuously exhibited high importance for communities located in the central area and showed stronger associations in spring and summer. This reflected that during periods when residents’ willingness to engage in outdoor activities increased, the accessibility of green spaces was of key significance for emotional health. In contrast, indicators such as the LPI exhibited relatively small seasonal fluctuations, indicating a more stable influence on emotions. Based on these findings, future urban green space planning should, while ensuring baseline greenness, improve residents’ actual accessibility and usability of green spaces in daily scenarios through measures such as optimizing road network structures, rationally configuring green space entrances and exits, and improving walking environments, may help improve the context sensitivity of planning.

Second, the spatial heterogeneity of key factors indicates that green space planning for emotional well-being should be tailored to contexts rather than relying on a uniform spatial strategy. The updated results indicate that NDVI, green space openness, and green space accessibility exhibit distinct spatial patterns in contributions to negative emotions. NDVI showed mixed contributions, with negative contributions widely distributed in many peripheral communities as well as some inner-city areas, while positive contributions were concentrated in several belt-like and clustered zones, particularly in parts of the central-southern and eastern areas. Green space accessibility displayed a clearer center–periphery contrast, with positive contributions concentrated in several inner-city and belt-like areas and negative contributions more widely distributed in the outer urban fringe. By contrast, green space openness showed an opposite tendency in many areas, with positive contributions more common in peripheral communities and negative contributions concentrated in parts of the urban core. These differences suggest that quantity-, access-, and openness-related dimensions of UGS may be associated with predicted negative emotions in different ways across communities. Accordingly, in areas where NDVI or landscape structure is more locally salient, protecting large green patches and avoiding excessive fragmentation may remain important, whereas in communities where accessibility- or openness-related factors are more relevant, improving walkable access, enhancing the usability of open green spaces, and providing smaller but more reachable green amenities may be more appropriate planning priorities (25, 55).

Finally, the seasonal results suggest that planning priorities may need to vary over time rather than remain fixed throughout the year. In the present study, NDVI showed relatively stable importance across all seasons, green space openness showed stronger associations in summer, and green space accessibility displayed more continuous positive associations in autumn. This implies that different dimensions of UGS may become more salient in different seasonal contexts. Accordingly, maintaining stable greenness may remain important throughout the year, while the accessibility and openness of green spaces may deserve greater attention during seasons in which outdoor use and environmental perception are more active. In practice, this may involve adjusting management priorities seasonally—for example, by strengthening pedestrian access, improving facilities for seasonal use, and maintaining open and usable green environments when residents are more likely to interact with them (34).

### 5.3 Limitations and prospects

This study still has several limitations. First, the social media data used here cannot fully represent the emotional experiences of the entire population. Sina Weibo users are more likely to be young and middle-aged, whereas older adults, children, and people who rarely use social media are underrepresented. Moreover, even after population standardization, the dependent variable still reflects the community-level spatial expression of negative emotions on social media rather than individual-level clinical emotional severity. Future studies could improve generalizability by combining social media with surveys, interviews, or community-level field investigations. Second, although this study incorporated lockdown intensity as a control variable, the analysis was still conducted within the socially distinctive context of Wuhan in 2022, when epidemic-control measures and mobility constraints had not fully disappeared. As a result, some residual contextual influence may still remain, and the findings should be generalized to other years with caution. Third, the factors associated with negative emotions are likely more complex than those included in this study. In addition to green space quantity, quality, and landscape pattern, subjective perceptions, environmental satisfaction, social status, and other contextual conditions may also play important roles. Future studies should consider integrating these dimensions and conducting cross-validation using multi-source data. Furthermore, because the seasonal analysis in this study covered only one year, it cannot fully capture longer-term interannual variation. Subsequent research could use multi-year data to more systematically characterize temporal changes in the association between UGS and emotional outcomes.

## 6 Conclusion

This study systematically examined the nonlinear relationships and spatial heterogeneity in the associations between UGS characteristics and negative emotions across different seasons. Comparative experiments showed that the GWRF model outperformed traditional RF and GWR methods in terms of performance, providing new methodological support for geospatial nonlinear modeling in the field of urban green space and negative emotions. The main conclusions are as follows:

1. Spatiotemporal characteristics of negative emotions. Temporally, negative emotions were strongest in winter and weakest in summer. Spatially, negative emotions exhibited a significant “core–periphery” structure, with areas with higher negative emotions mainly concentrated in the central area of Wuhan City and the southern urban fringe.
2. Key factors and seasonal variation. NDVI, green space accessibility, and green space openness were identified as key factors associated with negative emotions, while their relative importance varied across seasons. NDVI remained the dominant factor throughout the full year and in all four seasonal models, and green space accessibility showed stronger seasonal prominence in spring and summer.
3. Nonlinear relationships and threshold effects. Several UGS indicators exhibited clear nonlinear patterns and identifiable turning points, and some of these turning points were relatively comparable across seasons. However, the magnitudes and directions of these relationships varied seasonally, with more pronounced changes in spring and relatively milder changes in winter.
4. Spatial heterogeneity. NDVI, green space openness, and green space accessibility all exhibited substantial spatial heterogeneity in their associations with negative emotions, but their spatial patterns differed. NDVI and green space accessibility showed broadly similar spatial structures, whereas green space openness displayed a distinct pattern with more positive associations in peripheral communities and more negative associations in parts of the urban core. These results suggest that different dimensions of UGS may be associated with emotional outcomes through different spatial mechanisms.
5. Policy implications. The findings suggest the value of adopting a seasonally dynamic perspective in green space planning. In practice, this means identifying the locally dominant UGS-related factors in different seasons and taking into account both nonlinear patterns and spatial heterogeneity when prioritizing interventions, such an approach may help improve the context sensitivity of green space planning.

## Data Availability

The original contributions presented in the study are included in the article/supplementary material, further inquiries can be directed to the corresponding authors.
